# Comparative validation of PREDICT versions 3.1 and 2.2 for overall survival in the Dutch breast cancer population

**DOI:** 10.1016/j.breast.2025.104681

**Published:** 2025-12-16

**Authors:** Lara W.A. Vreven, Elfi M. Verheul, Marissa C. van Maaren, Frank Doornkamp, Robert-Jan Schipper, Sabine Siesling, Paul D.P. Pharoah, Vivianne C.G. Tjan-Heijnen, Adri C. Voogd

**Affiliations:** a Department of Epidemiology, Maastricht University, PO Box 616, 6200 MD, Maastricht, the Netherlands; b Department of Public Health, Centre for Medical Decision Making, Erasmus University Medical Centre, 3015 GD, Rotterdam, the Netherlands; c Scientific Bureau, Dutch Institute for Clinical Auditing, 2333 AA, Leiden, the Netherlands; d Department of Health Technology and Services Research, Technical Medical Centre, University of Twente, 7522 NH, Enschede, the Netherlands; e Department of Research and Development, Netherlands Comprehensive Cancer Organisation (IKNL), 3511 CV, Utrecht, the Netherlands; f Department of Biomedical Data Sciences, Leiden University Medical Centre, 2300 RC, Leiden, the Netherlands; g Department of Surgery, Catharina Hospital, 5623 EJ, Eindhoven, the Netherlands; h Department of Computational Biomedicine, Cedars-Sinai Medical Center, West Hollywood, CA 90069, USA; i Department of Medical Oncology, GROW Research Institute for Oncology and Reproduction, Maastricht University Medical Centre+, 6202 AZ, Maastricht, the Netherlands

**Keywords:** Breast cancer, Overall survival, Dutch population, PREDICT, Prediction model, Validation

## Abstract

**Background:**

PREDICT Breast is a clinical decision-support tool estimating prognosis and the absolute benefit of adjuvant systemic therapies in early breast cancer. PREDICT v2.2 is recommended in Dutch guidelines. Both v2.2 and the recently updated v3.1 have not been validated in the Dutch population. This study compares the predictive performance of PREDICT v3.1 and v2.2 for 10-year OS in Dutch breast cancer patients.

**Methods:**

Women diagnosed between 2005 and 2013 with primary invasive breast cancer were selected from the Netherlands Cancer Registry. Ten-year OS predictions from v2.2 and v3.1 were compared with observed OS for the overall cohort and 36 subgroups defined by oestrogen receptor (ER) status, HER2-status, age, and tumour stage. Discrimination (ability to distinguish patients with different outcomes) and calibration (agreement between predicted and observed outcomes) of both models were assessed.

**Results:**

Among 101,282 patients, both versions showed moderate discrimination (AUC v2.2 = 0.768; v3.1 = 0.775) and calibration (v2.2 intercept: 0.07; slope: 1.09; v3.1 intercept: 0.12, slope: 1.00). V3.1 slightly overestimated (1.9%), whereas v2.2 slightly underestimated (1.6%) 10-year OS. Across subgroups, v3.1 generally outperformed v2.2 except in patients aged >75 years, where v2.2 provided more accurate estimates. In ER-/HER- patients aged 50–75 years, v3.1 overestimated (1.5–2.8%) and v2.2 underestimated (2.8–5.3%) 10-year OS.

**Conclusion:**

Both PREDICT v2.2 and v3.1 accurately predict 10-year OS in Dutch breast cancer patients, with small differences between versions that vary by subgroup. No single model is optimal for all patients highlighting the need for subgroup-specific recalibration and careful interpretation when applying PREDICT.

## Highlights

• First Dutch validation comparing PREDICT Breast v2.2 and v3.1
• PREDICT Breast v3.1 shows improved calibration over v2.2 in most Dutch subgroups.
• PREDICT Breast v2.2 and v3.1 show moderate discrimination for 10-year survival.
• Subgroup-specific recalibration may improve clinical use of PREDICT Breast.
• All risk models, like genomic assays, have limitations and need population-specific validation.

## 1 Introduction

In Europe, approximately 600,000 patients were diagnosed with breast cancer in 2022 [1]. This highlights the need for accurate prediction tools to support personalized treatment decisions [2]. PREDICT Breast is a clinical prediction model that supports decision-making on adjuvant systemic therapy for women with early invasive breast cancer by predicting baseline survival and the added benefit of adjuvant systemic therapies on 5-, 10-, and 15-year overall survival (OS) based on tumour- and patient-related characteristics [3].

Genomic assays such as Oncotype DX and MammaPrint are widely used to guide chemotherapy decisions. They were originally developed and validated as prognostic tools estimating baseline risk of distant recurrence [[4], [5], [6]]. Accumulating data, however, suggests that these assays may also carry predictive information on chemotherapy benefit in hormone receptor-positive, HER2-negative early breast cancer [7,8]. At the same time, the ASTER study [9] and Beltran-Bless et al. [10] support that genomic scores often remain predominantly prognostic, and the extent to which they provide independent treatment-predictive information remains debated. This highlights the importance of validated clinical models like PREDICT and the integration of clinicopathological and genomic information.

The PREDICT algorithm, originally released in 2010 using eastern UK data from 1993 to 2003, estimates 5- and 10-year OS [3]. Since its initial release, PREDICT has been continuously updated to improve accuracy and incorporate new clinical advancements. V2.0 (2017) added predictors such as age at diagnosis, the exact tumour size, number of positive lymph nodes, and the presence of micrometastases, while subsequent updates (v2.1 and v2.2) included bisphosphonates therapy, 15-year OS estimates, and refined hormone therapy options [11]. V3.0 (2024) incorporated updated hazard ratios, progesterone receptor status, radiotherapy effects on breast cancer-specific mortality, and treatment effects on other causes of mortality [12], with v3.1, further refining baseline hazards for patients undergoing primary surgery.

The most recent validated version in the Dutch breast cancer population is PREDICT v2.0, which demonstrated good overall accuracy, indicating that predicted risks aligned reasonably well with observed outcomes and that the model could adequately distinguish between patients with different outcomes (e.g. alive vs dead). However, the model performed worse in oestrogen receptor (ER) negative patients, patients aged ≥75 years, T3 tumours, and those receiving both endocrine therapy and chemotherapy [13]. This supports the need to externally validate the updated version of PREDICT in predefined subgroups.

As PREDICT v2.2 is currently recommended in Dutch clinical guidelines [14], this study aims to evaluate whether PREDICT v3.1 provides improved predictive performance compared to v2.2 for 10-year OS in the Dutch breast cancer population. In addition, clinically relevant subgroups defined by ER status, HER2 status, age, and tumour stage were used to validate both PREDICT models.

## 2 Methods

### 2.1 Design

In this population-based external validation cohort study, data on patient-, tumour-, and treatment-related characteristics from the Netherlands Cancer Registry (NCR), which is hosted by the Netherlands Comprehensive Cancer Organisation (IKNL), were used. It is a comprehensive, population-based database that includes data on all newly diagnosed cancer cases in the Netherlands, registered by trained data managers. Included variables corresponded to those in PREDICT Breast: age, smoking status, ER status, progesterone status, HER2 status, Ki-67 status, tumour size and grade, mode of detection, and number of positive lymph nodes.

Tumour staging followed the tumour, node, and metastasis (TNM) classification system, with the use of the 6th or 7th edition depending on the year of diagnosis [15,16]. Chemotherapy classification changed from generation-based in v2.2 to standard-dose anthracycline-based and high-dose anthracycline and/or taxane-based regimes in v3.1. Vital status and date of death were derived from the Municipal Personal Records database, which contained complete records until February 2024. Because cause of death was not recorded, we analysed all-cause mortality without distinguishing between breast cancer-specific and other-cause mortality.

### 2.2 Patients

In this study, all women with primary invasive breast cancer in the Netherlands, diagnosed from 2005 to 2013, who underwent surgery were included. Patients who received neoadjuvant therapy, had no pathologically confirmed tumour, had metastatic disease at diagnosis, were younger than 25 or older than 85 years, or were recorded as alive but with incomplete follow-up information were excluded. These inclusion criteria match those of the development cohorts of PREDICT v2.2 and v3.1 [3,12].

### 2.3 Statistical analysis

The outcome of interest was 10-year OS, defined as time from diagnosis to death from any cause, last observation or end of follow-up. Predicted OS was calculated using PREDICT v2.2 and v3.1 functions obtained from the developer (P.D.P.P). We compared the 10-year predicted survival probabilities with the observed 10-year survival in the data using Kaplan-Meier estimates.

For missing data, the PREDICT algorithm allows specific variables to be missing (Ki-67 status, progesterone status and mode of detection). For the other variables we used multiple imputation (MICE) [17] with 5 imputations for tumour size, number of positive lymph nodes, differentiation grade, HER2 status, and ER status. A single dataset was created by averaging numeric variables and taking the majority for categorical variables. Smoking status was unavailable for 101,207 patients (>99.9%) in the validation cohort, therefore smoking status was coded as non-smoking for the entire cohort.

Calibration (agreement between predicted and observed outcomes) and discrimination (the ability to distinguish between patients with different outcomes, e.g. alive vs dead) were assessed [18] for the total cohort and 36 subgroups based on ER status (positive or negative), HER2 status (positive or negative), age (<50, 50–75 or >75), and TNM stage (I, II or III). A 95% confidence interval was calculated for observed OS based on Kaplan Meier estimates. Confidence intervals for the predictions were not computed, as PREDICT does not consider uncertainties in its predictions.

Simplified calibration plots were used to visually assess how well model predictions align with the observed outcome frequencies. Calibration slope was used to assess over- or underfitting. A slope <1 indicates overfitting: small risks are underestimated, and large risks are overestimated, while a slope >1 indicates the opposite (underfitting). If the slope equals 1 but the intercept differs from 0, predictions are systemically too high (intercept <0) or too low (intercept >0) on average [19].

Discriminative accuracy, which reflects the model's ability to distinguish between different outcomes (i.e. dead vs alive at 10 years), was assessed using the area under the receiver operating curve (AUC). An AUC closer to 1.0 indicates excellent discrimination, while a value of 0.5 suggests no predictive capability beyond random chance. AUC values were interpreted as follows: ≥0.80 is good, 0.69–0.79 is moderate, and <0.69 is poor [20].

A subgroup analysis was performed for patients who did not receive adjuvant therapy, representing the baseline on which adjuvant treatment decisions are made. Calibration (agreement between predicted and observed outcomes) and discrimination (the ability to distinguish between patients with different outcomes) were assessed [18]. All statistical analyses were performed in R version 4.4.2. Validation and reporting were carried out according to the TRIPOD criteria (Supplementary Table 1) [21].

## 3 Results

### 3.1 Patient characteristics

**Fig. 1:**
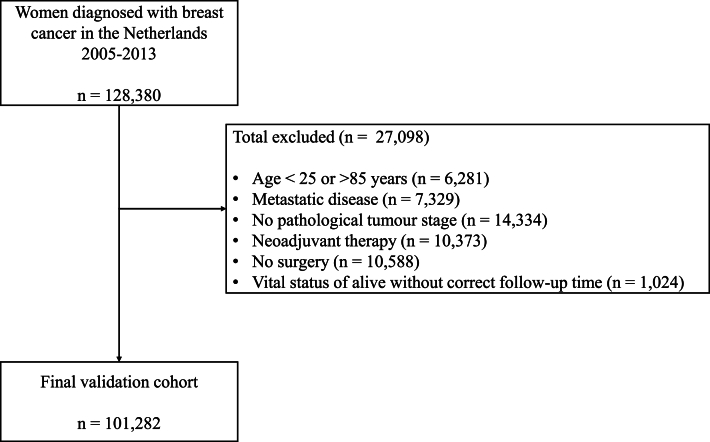
Patient selection for the Dutch study cohort for the validation of PREDICT v2.2 and v3.1. Flowchart illustrating patient selection for the Dutch study cohort used in the validation of PREDICT v2.2 and v3.1, including exclusion criteria and final cohort count.

A total of 128,380 women with primary invasive breast cancer diagnosed between 2005 and 2013 were identified from the NCR (Fig. 1). In total 101,282 (78.9%) patients met eligibility criteria (Fig. 1; Table 1). Adjuvant therapy use differed between subgroups, with chemotherapy and trastuzumab most common in young HER2+ patients, and hormone therapy in ER+ subgroups (Supplementary Table 2). Patients not receiving adjuvant therapy were older and had higher 10-year survival compared with the total population (Supplementary Table 3).

**Table 1:** Baseline characteristics, treatment and 10-year survival in the Dutch validation cohort.

Characteristic			n = 101,282
Year of diagnosis			2005-2013
Age, median (IQR)			60.0 (51.0–69.0)
Menopausal status, n (%)^a^			
	Pre-menopausal		7874 (23.9)
	Post-menopausal		25,136 (76.1)
	Missing		68,272
Tumour size (mm), median (IQR)			16.0 (11.0–23.0)
	Missing		2170
Lymph nodes, n (%)			
	≥ 1		34,377 (34.4)
	0		65,470 (65.6)
	Missing		1435
Tumour stage, n (%)			
	Stage I		52,969 (52.3)
	Stage II		38,233 (37.7)
	Stage III		10,080 (10.0)
Differentiation, n (%)			
	Grade 1		24,193 (24.9)
	Grade 2		44,155 (45.5)
	Grade 3		29,792 (29.6)
	Missing		4142
Oestrogen receptor status, n (%)			
	Positive		83,804 (83.9)
	Negative		16,054 (16.1)
	Missing		1424
HER2 status, n (%)			
	Positive		12,265 (12.1)
	Negative		83,713 (82.9)
	Unknown		5039 (5.0)
	Missing		265
Progesterone status, n (%)			
	Positive		66,467 (65.8)
	Negative		30,974 (30.7)
	Unknown		3563 (3.5)
	Missing		278
Mode of detection, n (%)			
	Clinically detected		12,417 (44.3)
	Screening detected		15,620 (55.7)
	Missing		73,245
Adjuvant therapy, n (%)			
	Chemotherapy		38,498 (38.0)
		Generation 2^b^	13,738 (13.6)
		Generation 3^b^	24,757 (24.4)
	Hormone therapy		51,737 (51.1)
	Trastuzumab		7684 (7.6)
	Radiotherapy		67,045 (66.2)
Vital status at 10-year, n (%)			
	Alive		77,739 (76.8)
	Death		23,543 (23.2)

Abbreviations: IQR = interquartile range, HER2 = human epidermal growth factor receptor 2.

a Menopausal status was used to determine eligibility for bisphosphonate therapy and was not included as predictor in the model.

b v3.1: Generation 2 = standard-dose anthracycline; Generation 3 = high-dose anthracycline/taxane-based.

### 3.2 Discriminatory accuracy and ability for 10-year overall survival

The discriminatory accuracy for 10-year OS was moderate in the total population for both PREDICT versions. The AUC of v2.2 was 0.768 (95% CI: 0.765–0.771), while for v3.1 it was slightly higher at 0.775 (95% CI: 0.772–0.778). The difference in AUCs was 0.007 (95% CI: 0.006–0.008), indicating a small but consistent improvement in discriminatory performance with v3.1.

A similar pattern was observed in patients not receiving adjuvant therapy, with an AUC of 0.818 (95% CI: 0.811–0.825) for v2.2 and 0.826 (95% CI: 0.818–0.833) for v3.1.

### 3.3 Calibration for 10-year overall survival

**Fig. 2:**
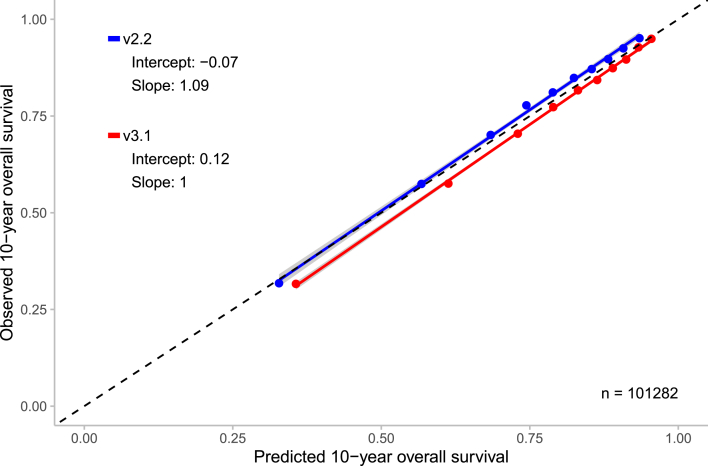
Observed and predicted 10-year overall survival for the Dutch validation cohort of PREDICT v2.2 and v3.1 with the 95% confidence interval. n = number of patients included in the external validation set. Calibration is assessed using the intercept (ideally 0) and slope (ideally 1). The Y-axis represents the observed 10-year overall survival, while the X-axis represents the predicted 10-year overall survival. Each dot indicate the average for a decile of predicted probabilities. In grey, the 95% confidence intervals are shown.

At 10-year, v3.1 slightly overestimated OS in the total cohort, with a mean predicted survival of 78.7% compared to an observed survival of 76.8% (difference: +1.9%; Table 2). In contrast, v2.2 slightly underestimated OS with a predicted survival of 75.2% (difference: -1.6%; Table 2). Calibration plots for the total cohort demonstrated good agreement between predicted and observed OS for both models, with slopes of 1.09 (v2.2) and 1.00 (v3.1) and intercepts of −0.07 and 0.12, respectively (Fig. 2).

**Table 2:** Observed and predicted 10-year overall survival by subgroups based on ER status, HER2 status, age, and tumour stage for PREDICT v2.2 and v3.1.

Total cohort						PREDICT v2.2		PREDICT v3.1	
				N (%)	Observed, % (95% CI)	Predicted, %	Difference, %	Predicted, %	Difference, %
				101282 (100.0)	76.8 (76.5–77.0)	75.2	−1.6	78.7	1.9
**ER-status**	**HER2-status**	**Age**	**Stage**						
ER+	HER2+	<50	I	877 (0.9)	94.4 (92.9–95.9)	90.5	−3.9	92.7	−1.7
			II	1163 (1.1)	91.1 (89.4–92.7)	84.9	−6.2	87.9	−3.2
			III	424 (0.4)	79.0 (75.2–83.0)	59.9	−19.1	69.1	−9.9
		50–75	I	2422 (2.4)	84.6 (83.1–86.0)	82.1	−2.4	85.4	0.9
			II	1849 (1.8)	78.7 (76.9–80.6)	76.4	−2.3	79.1	0.4
			III	546 (0.5)	65.9 (62.1–70.0)	54.8	−11.1	61.0	−5.0
		>75	I	153 (0.1)	46.4 (39.1–55.0)	46.9	0.5	51.5	5.1
			II	323 (0.3)	31.0 (26.3–36.4)	37.8	6.9	39.4	8.4
			III	114 (0.1)	20.2 (14.0–29.1)	21.0	0.9	22.4	2.2
									
ER+	HER2-	<50	I	6502 (6.4)	94.2 (93.6–94.8)	92.2	−2.0	94.0	−0.2
			II	6354 (6.3)	89.3 (88.6–90.1)	87.4	−1.9	89.5	0.2
			III	1748 (1.7)	73.4 (71.4–75.5)	67.1	−6.3	73.9	0.5
		50–75	I	33331 (32.9)	84.6 (84.2–5.0)	82.9	−1.6	86.8	2.2
			II	17175 (17.0)	78.45 (77.8–79.1)	78.6	0.2	81.7	3.2
			III	3972 (3.9)	60.8 (59.3–62.3)	60.2	−0.5	65.4	4.6
		>75	I	3062 (3.0)	44.4 (42.7–46.2)	48.9	4.5	54.6	10.2
			II	4068 (4.0)	33.8 (32.3–35.2)	42.6	8.8	46.0	12.2
			III	1020 (1.0)	21.2 (18.8–23.8)	27.6	6.4	29.3	8.1
									
ER-	HER2+	<50	I	353 (0.3)	90.9 (88.0–94.0)	83.1	−7.8	86.5	−4.4
			II	537 (0.5)	85.3 (82.3–88.3)	75.7	−9.5	76.0	−9.3
			III	297 (0.3)	69.0 (64.0–74.5)	47.8	−21.2	48.5	−20.6
		50–75	I	1227 (1.2)	84.5 (82.5–86.6)	72.2	−12.3	80.3	−4.2
			II	1216 (1.2)	78.0 (75.8–80.4)	65.3	−12.7	69.8	−8.2
			III	509 (0.5)	64.6 (60.6–68.9)	37.9	−26.8	44.6	−20.1
		>75	I	87 (0.1)	28.7 (20.6–40.0)	35.4	6.7	38.8	10.1
			II	213 (0.2)	30.5 (24.9–37.4)	26.1	−4.4	23.6	−6.9
			III	120 (0.1)	8.3 (4.6–15.1)	7.6	−0.7	6.5	−1.8
									
ER-	HER2-	<50	I	1367 (1.4)	89.9 (88.3–91.5)	84.3	−5.6	86.2	−3.7
			II	1830 (1.8)	80.3 (78.5–82.2)	76.7	−3.6	75.7	−4.6
			III	373 (0.4)	52.8 (48.0–58.1)	46.1	−6.7	47.1	−5.7
		50–75	I	3286 (3.2)	78.1 (76.7–79.5)	73.3	−4.8	80.9	2.8
			II	2795 (2.8)	67.7 (66.0–69.5)	65.0	−2.8	69.2	1.5
			III	697 (0.7)	40.9 (37.4–44.7)	35.6	−5.3	42.0	1.1
		>75	I	302 (0.3)	31.5 (26.6–37.2)	41.0	9.5	45.9	14.4
			II	710 (0.7)	26.1 (23.0–29.5)	32.1	6.1	31.6	5.6
			III	260 (0.3)	11.9 (8.6–16.6)	32.3	0.5	11.4	−0.6

Abbreviations: N = total number, CI = confidence interval, ER = oestrogen receptor, HER2 = human epidermal growth factor receptor 2.

**Fig. 3:**
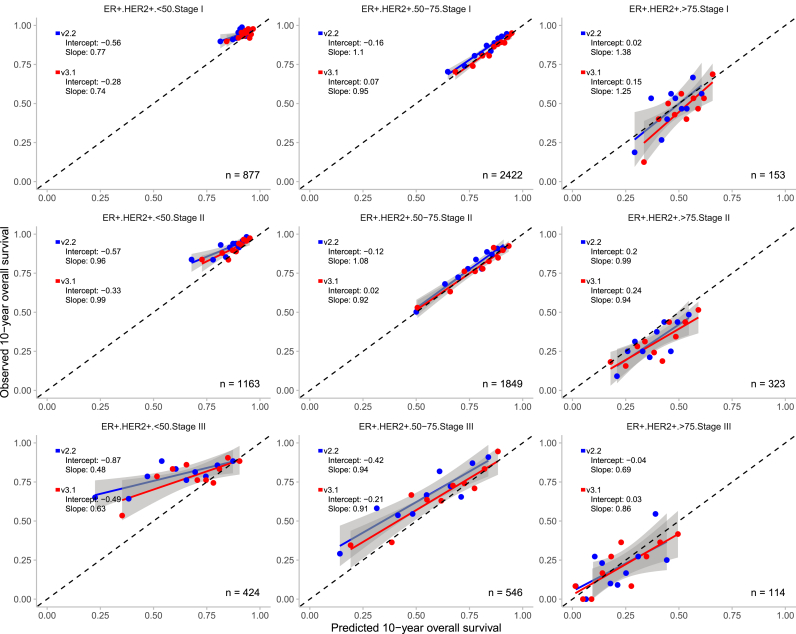
Observed and predicted 10-year overall survival for patients with oestrogen receptor (ER) positive and human epidermal growth factor receptor 2 (HER2) positive breast cancer, stratified by age and tumour stage with the 95% confidence interval. n = number of patients included in the external validation set. Calibration is assessed using the intercept (ideally 0) and slope (ideally 1). The Y-axis represents the observed 10-year overall survival, while the X-axis represents the predicted 10-year overall survival. Each dot indicate the average for a decile of predicted probabilities. In grey, the 95% confidence intervals are shown.

**Fig. 4:**
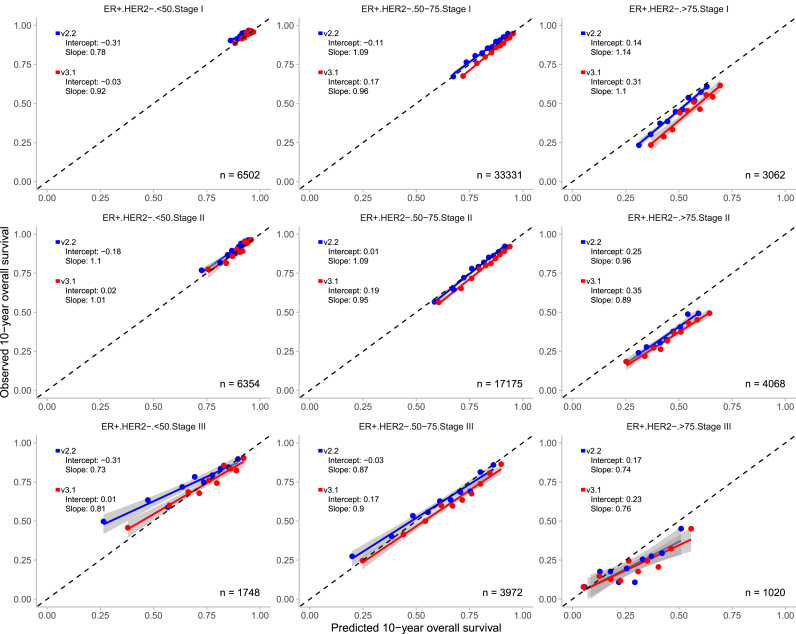
Observed and predicted 10-year overall survival for patients with oestrogen receptor (ER) positive and human epidermal growth factor receptor 2 (HER2) negative breast cancer, stratified by age and tumour stage with the 95% confidence interval. n = number of patients included in the external validation set. Calibration is assessed using the intercept (ideally 0) and slope (ideally 1). The Y-axis represents the observed 10-year overall survival, while the X-axis represents the predicted 10-year overall survival. Each dot indicate the average for a decile of predicted probabilities. In grey, the 95% confidence intervals are shown.

**Fig. 5:**
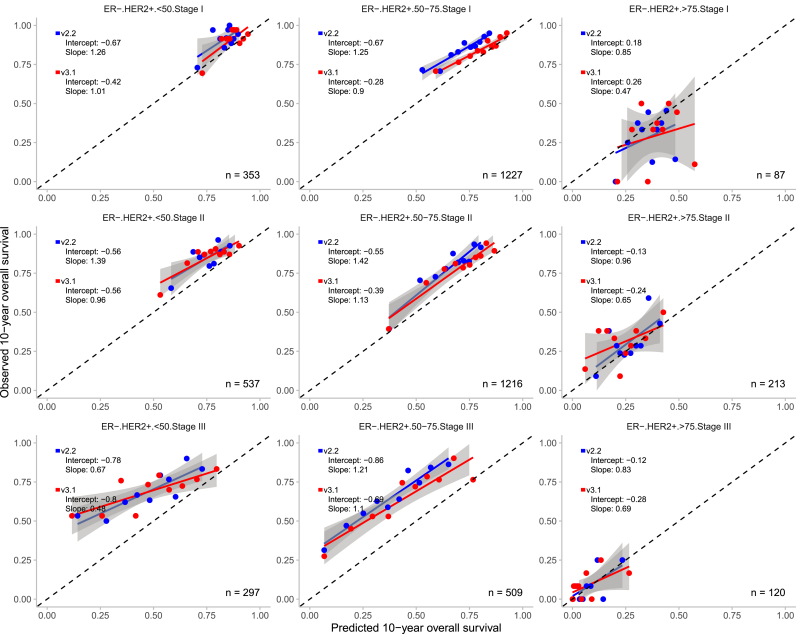
Observed and predicted 10-year overall survival for patients with oestrogen receptor (ER) negative and human epidermal growth factor receptor 2 (HER2) positive breast cancer, stratified by age and tumour stage with the 95% confidence interval. n = number of patients included in the external validation set. Calibration is assessed using the intercept (ideally 0) and slope (ideally 1). The Y-axis represents the observed 10-year overall survival, while the X-axis represents the predicted 10-year overall survival. Each dot indicate the average for a decile of predicted probabilities. In grey, the 95% confidence intervals are shown.

**Fig. 6:**
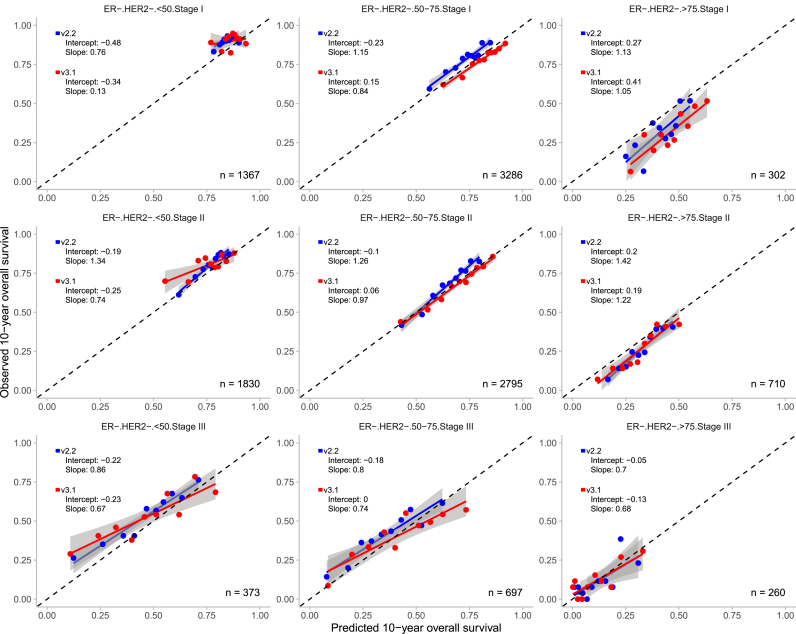
Observed and predicted 10-year overall survival for patients with oestrogen receptor (ER) negative and human epidermal growth factor receptor 2 (HER2) negative breast cancer, stratified by age and tumour stage with the 95% confidence interval. n = number of patients included in the external validation set. Calibration is assessed using the intercept (ideally 0) and slope (ideally 1). The Y-axis represents the observed 10-year overall survival, while the X-axis represents the predicted 10-year overall survival. Each dot indicate the average for a decile of predicted probabilities. In grey, the 95% confidence intervals are shown.

Overall, both PREDICT models demonstrated good calibration across the 36 predefined subgroups, with calibration slopes close to 1 and intercepts close to 0 in the majority of the cases (Fig. 3, Fig. 4, Fig. 5, Fig. 6). V3.1 demonstrated better calibration for 10-year OS than v2.2 in the majority of the subgroups (Table 2).

Among ER-/HER2- patients aged 50–75 years, the models demonstrated opposite calibration patterns across all tumour stages, which was similar to the calibration observed in the overall population. V3.1 tended to overestimate 10-year OS by 1.5%–2.8%, whereas v2.2 underestimated survival by 2.8%–5.3%.

However, notable exceptions were observed. In certain subgroups v2.2 outperformed v3.1. In patients aged >75 years, v3.1 tended to overestimate survival compared with v2.2. For example, in ER+/HER2- patients with stage II disease, v3.1 overestimated survival more by 12.2% versus 8.8% with v2.2. Similarly, in ER-/HER2-patients with stage I disease, overestimation was 14.4% for v3.1 compared to 9.5% for v2.2. Comparable patterns were noted in ER+/HER2+ patients.

For patients not treated with adjuvant therapy, both versions overestimated 10-year OS, with 4.1% (v2.2) and 6.7% (v3.1) (Supplementary Table 4). The calibration plot shows good agreement between predicted and observed survival, with slopes of 1.09 (v2.2) and 0.96 (v3.1) and intercepts of 0.21 and 0.33, respectively (Supplementary Fig. 1).

## 4 Discussion

In this study, we compared the performance of PREDICT v3.1 and PREDICT v2.2 in an external validation predicting 10-year OS in a large Dutch breast cancer cohort. Both versions demonstrated moderate discriminatory accuracy (the ability to distinguish between patients with different outcomes) and good calibration (agreement between predicted and observed outcomes) in the overall population. V3.1 slightly overestimated OS by 1.9%, while v2.2 underestimated OS by 1.6%. Across the subgroups v3.1 outperformed v2.2 in the majority of the 36 predefined subgroups, although notable exceptions were observed. In older patients, aged >75 years, v2.2 tended to have more accurate predictions than v3.1, particularly in ER+/HER2- patients. Among ER-/HER2- patients aged 50–75 years, the models showed opposite calibration trends with v3.1 overestimating and v2.2 underestimating survival. These patterns suggest that while both versions are generally well-calibrated, certain patient populations may benefit from tailored recalibration. PREDICT estimates both prognosis and the benefit of adjuvant systemic therapies, providing accessible and complementary clinical value to primarily prognostic genomic assays. Just like these assays, PREDICT has limitations in certain subgroups, emphasizing the need for ongoing validation and careful interpretation in clinical practice.

Compared to previous PREDICT validations, we observed similar discriminatory performance, with AUC comparable with earlier studies worldwide (0.709–0.780) reported for various PREDICT versions [13,[22], [23], [24]].

While PREDICT consistently demonstrates moderate discrimination, calibration is more important than discrimination for absolute risk estimation [25]. V2.2 showed slightly better calibration than v3.1 for 10-year OS in the overall Dutch cohort. Consistent with our findings, the Dutch validation study of PREDICT v2.0 [13], using patients diagnosed in 2005, reported a 10-year OS difference of −1.0% compared with −1.6% (v2.2) and 2.0% (v3.1) in our study, suggesting that all versions performed well across the Dutch population. Chen et al. [24] found improved calibration for v3.0 compared to v2.2 in a Chinese cohort for the 5-year OS. Direct comparisons are challenging due to differences in model versions, survival outcomes (5 vs. 10 years), healthcare systems, and patient populations. Availability and uptake of (insured) treatment options differ across countries. For instance, trastuzumab was rapidly implemented after approval in the Netherlands [26], while uptake was slower elsewhere [27], influencing validation outcomes. Moreover, although PREDICT v3.1 distinguishes standard-from high-dose anthracycline regimens, high-dose anthracyclines are now mostly reserved for high-risk patients. Therefore, this distinction is of limited relevance for most contemporary patients.

Calibration in specific subgroups indicated lower predictive accuracy for patients aged >75 years and ER- tumours, in combination with other tumour characteristics. These findings are consistent with validation studies of earlier PREDICT versions [13,24,28] and may reflect the underrepresentation of these patients implying that caution remains warranted. In contrast, v3.1 showed clear improvement for patients <75 years and HER2+ tumours, for whom v2.2 underestimated OS. Chen et al. previously reported improved accuracy in v3.0 for this subgroup [24], reflecting updated treatment effects that capture improved prognosis associated with advances in HER2-targeted therapy [29]. Accordingly, the improved calibration in v3.1 for HER2+ patients aligns with these updates. Nevertheless, although the lower estimated benefit of various adjuvant systemic treatments [12] may reflect updated evidence, careful interpretation is necessary to prevent overly conservative decisions that might inadvertently lead to undertreatment and compromise long-term outcomes. In patients not receiving any adjuvant systemic therapy, both versions showed good discrimination but overestimated OS, which may indicate patient selection.

Predictions for high-risk patients, such as those with HER2+ tumours or advanced tumour stage, often deviated from observed survival. Clinically, however, this is less relevant, as these patients almost invariably receive adjuvant treatment regardless of predicted risk. Calibration was considerably better in low- or intermediate-risk patients, where predictions more closely aligned with observed outcomes and may therefore support clinical decision-making for adjuvant therapy [30].

Differences and similarities between our Dutch cohort and the UK development dataset were observed [12]. Age, tumour size, tumour grade, and prevalence of ER-positive disease were largely comparable, whereas treatment patterns differed more notably. Only 51.1% of patients in our cohort received endocrine therapy, compared with 60% in eastern UK and 40% in West Midlands [12]. Differences in endocrine therapy uptake may affect calibration, as PREDICT incorporates treatment benefit. Additional factors, including patient preferences, shared decision-making, and limitations in registry-based data not capturing treatment adherence may contribute to cohort differences.

Patients who received neoadjuvant therapy are excluded in PREDICT. The use of neoadjuvant therapy in the Netherlands has increased over time from 9% in 2005 to 51% in 2023 [31], meaning that patients who still undergo upfront surgery, as in our cohort, represent a selected subgroup that no longer reflects routine clinical care in 2025. Although survival is expected to be similar in patients receiving neoadjuvant therapy, different predictive factors may be required, as seen in the INFLUENCE model [32]. Therefore, future validation studies of PREDICT in neoadjuvant treated patients are necessary to determine how these patients can be accurately integrated into the PREDICT tool. Additionally, this selection bias may contribute to the observed overestimation of survival in subgroups that are now more frequently treated with neoadjuvant therapy and are therefore partly excluded from validation.

Calibration and discrimination of both PREDICT models were generally adequate at the population level. However, caution is warranted for certain subgroups. For instance, older patients and certain tumour subtypes may benefit from recalibration or careful interpretation of predictions. Tools such as PORTRET, developed for older patients, address limitations of commonly used models in this group [33]. These findings highlight the need to consider subgroup-specific performance when applying PREDICT in clinical practice.

### 4.1 Strengths and limitations

This is the first study to compare PREDICT v2.2 and v3.1 in estimating 10-year OS using a large, population-based Dutch cohort. A major strength of this study is the large sample size, enabling the stratification across clinically relevant subgroups, which gives a more nuanced evaluation of model performance and provides concrete recommendations based on patient and tumour characteristics.

Although our sample size was large, some subgroups (particularly patients aged >75 years and ER-/HER2+ patients aged <50 years) remained small. Results for these subgroups should be interpreted with caution. As seen in many prediction models, performance often decreases at the extremes, and preferably we would have had larger samples for more stable results [34]. However, their inclusion is crucial for the model's applicability for these subgroups.

Our findings should be interpreted in the context of contemporary clinical practice. While PREDICT is widely used, its limitations are most evident in aggressive tumour subtypes, extreme age groups, and neoadjuvant settings. Like genomic assays, model performance may vary across time and populations, highlighting the need for ongoing validation and careful interpretation.

Additionally, some assumptions were necessary as not all data was available. Smoking status was unavailable, however coding all patients as smokers versus non-smokers had minor effect on the results, therefore all patients were classified as non-smokers. Moreover, information on Ki-67 status was completely missing in our data and was therefore set to ‘unknown’. Nevertheless, improvements in predictive performance can be expected if information on Ki-67 is known [35]. Heart dose (in grays) from radiotherapy was missing and therefore assumed to be zero, despite variation across Dutch institutions (0.80–1.82 Gy) [36], as doses <2.5 Gy ensure adequate heart protection [37]. Bisphosphonate use was set to ‘not used’, although recommended for postmenopausal women receiving adjuvant systemic therapy, as it modestly improves OS [38]. However, this assumption is unlikely to bias results towards worse outcomes. In PREDICT, the duration of hormone therapy can be chosen to be 5 or 10 years. However, we did not have information about the duration of hormone therapy in the dataset and therefore we assumed a duration of 5-years for every patient, following the Dutch guidelines [39].

## 5 Conclusion

Both PREDICT v3.1 and v2.2 demonstrate moderate overall discrimination and good calibration for predicting 10-year OS in the Dutch breast cancer population. Within subgroups, differences in calibration between versions are minimal but vary across subgroups, indicating that no single model is optimal for all patient groups. These findings suggest that neither model consistently outperforms the other across all subgroups, highlighting the need for subgroup-specific recalibration and caution when interpreting predictions, particularly in subgroups where model's performance may be limited. It should be noted that primary validation was performed in the Dutch population. Further studies and integration of additional datasets are needed to assess the relevance and generalizability of PREDICT and improve predictive accuracy across diverse populations.

## CRediT authorship contribution statement

**Lara W.A. Vreven:** Writing – original draft, Visualization, Methodology, Formal analysis, Data curation. **Elfi M. Verheul:** Writing – review & editing, Formal analysis. **Marissa C. van Maaren:** Writing – review & editing. **Frank Doornkamp:** Writing – review & editing, Formal analysis. **Robert-Jan Schipper:** Writing – review & editing. **Sabine Siesling:** Writing – review & editing. **Paul D.P. Pharoah:** Writing – review & editing, Software. **Vivianne C.G. Tjan-Heijnen:** Writing – review & editing, Supervision, Conceptualization. **Adri C. Voogd:** Writing – review & editing, Supervision, Conceptualization.

## Data availability statement

We used data from the Netherlands Cancer Registry (NCR). This data is owned by the Netherlands Comprehensive Cancer Organisation (IKNL) and is available upon reasonable request.

## Declaration of generative AI and AI-assisted technologies in the writing process

During the preparation of this work the author(s) used ChatGPT in order to optimize the readability of the text. After using this tool/service, the author(s) reviewed and edited the content as needed and take(s) full responsibility for the content of the publication.

## Funding

This research did not receive any specific grant from funding agencies in the public, commercial, or not-for-profit sectors.

## Declaration of competing interest

The authors declare the following financial interests/personal relationships which may be considered as potential competing interests: Robert-JanSchipper reports a relationship with Catharina Hospital Eindhoven that includes: consulting or advisory.v2.

Given their role as specialty editor: epidemiology, Sabine Siesling had no involvement in the peer-review of this article and has no access to information regarding its peer-review. Full responsibility for the editorial process for this article was delegated to another journal editor.

Paul D.P. Pharoah receives a share of the fees received by Cambridge Enterprise for the licensing of PREDICT Breast to commercial partners.

If there are other authors, they declare that they have no known competing financial interests or personal relationships that could have appeared to influence the work reported in this paper.
