# Supplementary material for: Comparative validation of PREDICT versions 3.1 and 2.2 for overall survival in the Dutch breast cancer population
Source: Breast. 2025 Dec 16;85:104681. doi: 10.1016/j.breast.2025.104681 (PMC12771499; doi:10.1016/j.breast.2025.104681)
Supplement: Multimedia component 1 [file mmc1.docx]

**Supplementary material**

**Supplementary Table 1:** TRIPOD checklist

| **Section/Topic** | **Item** | **Checklist Item** | **Page** |
| --- | --- | --- | --- |
| **Title and abstract** | | | |
| Title | 1 | Identify the study as developing and/or validating a multivariable prediction model, the target population, and the outcome to be predicted. | Title |
| Abstract | 2 | Provide a summary of objectives, study design, setting, participants, sample size, predictors, outcome, statistical analysis, results, and conclusions. | Abstract |
| **Introduction** | | | |
| Background and objectives | 3a | Explain the medical context (including whether diagnostic or prognostic) and rationale for developing or validating the multivariable prediction model, including references to existing models. | 1. Introduction |
|  | 3b | Specify the objectives, including whether the study describes the development or validation of the model or both. | 1. Introduction |
| **Methods** | | | |
| Source of data | 4a | Describe the study design or source of data (e.g., randomized trial, cohort, or registry data), separately for the development and validation data sets, if applicable. | 2.1 Design |
|  | 4b | Specify the key study dates, including start of accrual; end of accrual; and, if applicable, end of follow-up. | 2.1 Design |
| Participants | 5a | Specify key elements of the study setting (e.g., primary care, secondary care, general population) including number and location of centres. | 2.1 Design |
|  | 5b | Describe eligibility criteria for participants. | 2.2 Patients |
|  | 5c | Give details of treatments received, if relevant. | N/A |
| Outcome | 6a | Clearly define the outcome that is predicted by the prediction model, including how and when assessed. | 2.3 Statistical analysis |
|  | 6b | Report any actions to blind assessment of the outcome to be predicted. | 2.3 Statistical analysis |
| Predictors | 7a | Clearly define all predictors used in developing or validating the multivariable prediction model, including how and when they were measured. | 2.1 Design |
|  | 7b | Report any actions to blind assessment of predictors for the outcome and other predictors. | 2.3 Statistical analysis |
| Sample size | 8 | Explain how the study size was arrived at. | 2.1 Design |
| Missing data | 9 | Describe how missing data were handled (e.g., complete-case analysis, single imputation, multiple imputation) with details of any imputation method. | 2.3 Statistical analysis |
| Statistical analysis methods | 10c | For validation, describe how the predictions were calculated. | 2.3 Statistical analysis |
|  | 10d | Specify all measures used to assess model performance and, if relevant, to compare multiple models. | 2.3 Statistical analysis |
|  | 10e | Describe any model updating (e.g., recalibration) arising from the validation, if done. | N/A |
| Risk groups | 11 | Provide details on how risk groups were created, if done. | 2.3 Statistical analysis |
| Development vs. validation | 12 | For validation, identify any differences from the development data in setting, eligibility criteria, outcome, and predictors. | 2.1 Design  2.2 Patients |
| **Results** | | | |
| Participants | 13a | Describe the flow of participants through the study, including the number of participants with and without the outcome and, if applicable, a summary of the follow-up time. A diagram may be helpful. | 3.1 Patient characteristics |
|  | 13b | Describe the characteristics of the participants (basic demographics, clinical features, available predictors), including the number of participants with missing data for predictors and outcome. | 3.1 Patient characteristics |
|  | 13c | For validation, show a comparison with the development data of the distribution of important variables (demographics, predictors and outcome). | 4. Discussion |
| Model performance | 16 | Report performance measures (with CIs) for the prediction model. | 3.2 Discriminatory  3.3 Calibration |
| Model-updating | 17 | If done, report the results from any model updating (i.e., model specification, model performance). | N/A |
| **Discussion** | | | |
| Limitations | 18 | Discuss any limitations of the study (such as nonrepresentative sample, few events per predictor, missing data). | 4.1 Limitations |
| Interpretation | 19a | For validation, discuss the results with reference to performance in the development data, and any other validation data. | 4. Discussion |
|  | 19b | Give an overall interpretation of the results, considering objectives, limitations, results from similar studies, and other relevant evidence. | 5. Conclusion |
| Implications | 20 | Discuss the potential clinical use of the model and implications for future research. | 5. Conclusion |
| **Other information** | | | |
| Supplementary information | 21 | Provide information about the availability of supplementary resources, such as study protocol, Web calculator, and data sets. | Data availability statement |
| Funding | 22 | Give the source of funding and the role of the funders for the present study. | Funding |

**Supplementary Table 2**. Distribution of administered treatments used by subgroups based on ER status, HER2 status, age, and tumour stage.

|  | | | |  | **Chemotherapy, n (%)** |  |  | **Hormone therapy, n (%)** | **Trastuzumab, n (%)** | **Radiotherapy, n (%)** |
| --- | --- | --- | --- | --- | --- | --- | --- | --- | --- | --- |
|  | | | |  |  | Generation 2, n (%) * | Generation 3, n (%) * |  |  |  |
| **ER-status** | **HER2-status** | **Age** | **Stage** | N (%) |  |  |  |  |  |  |
| ER+ | HER2+ | < 50 | I | 866 (0.9) | 596 (68.8) | 78 (9.0) | 518 (59.8) | 584 (67.4) | 563 (65.0) | 537 (63.0) |
|  |  |  | II | 1154 (1.1) | 1113 (96.4) | 145 (12.6) | 968 (83.9) | 1025 (88.8) | 1053 (91.2) | 647 (56.1) |
|  |  |  | III | 420 (0.4) | 414 (98.6) | 40 (9.5) | 374 (89.0) | 382 (91.0) | 391 (93.1) | 389 (92.9) |
|  |  | 50-75 | I | 2376 (2.3) | 811 (34.1) | 110 (4.6) | 701 (29.5) | 1272 (53.5) | 830 (34.9) | 1608 (67.7) |
|  |  |  | II | 1823 (1.8) | 1265 (69.4) | 182 (10) | 1082 (59.4) | 1610 (88.3) | 1201 (65.9) | 1005 (55.1) |
|  |  |  | III | 537 (0.5) | 441 (82.1) | 50 (9.3) | 391 (72.8) | 473 (88.1) | 427 (79.5) | 493 (91.8) |
|  |  | >75 | I | 152 (0.2) | 1 (0.7) | 0 (0.0) | 1 (0.7) | 85 (55.9) | 1 (0.7) | 54 (35.5) |
|  |  |  | II | 316 (0.3) | 2 (0.6) | 1 (0.3) | 1 (0.3) | 288 (91.1) | 8 (2.5) | 92 (29.1) |
|  |  |  | III | 112 (0.1) | 1 (0.9) | 0 (0.0) | 1 (0.9) | 104 (92.9) | 9 (8.0) | 82 (73.2) |
|  |  |  |  |  |  |  |  |  |  |  |
| ER+ | HER2- | < 50 | I | 6336 (6.3) | 2169 (34.2) | 938 (14.8) | 1231 (19.4) | 2812 (44.4) | 23 (0.4) | 4240 (66.9) |
|  |  |  | II | 6304 (6.2) | 5635 (89.4) | 2030 (32.2) | 3605 (57.2) | 5718 (90.7) | 36 (0.6) | 3758 (59.6) |
|  |  |  | III | 1744 (1.7) | 1692 (97.0) | 396 (22.7) | 1296 (74.3) | 1625 (93.2) | 14 (0.8) | 1632 (93.6) |
|  |  | 50-75 | I | 32587 (32.2) | 2949 (9.0) | 1341 (4.1) | 1608 (4.9) | 10494 (32.2) | 46 (0.1) | 24529 (75.3) |
|  |  |  | II | 17035 (16.8) | 7922 (46.5) | 3343 (19.6) | 4578 (26.9) | 15195 (89.2) | 53 (0.3) | 10160 (59.6) |
|  |  |  | III | 3960 (3.9) | 3006 (75.9) | 1107 (28.0) | 1899 (48) | 3707 (93.6) | 17 (0.4) | 3582 (90.5) |
|  |  | >75 | I | 3009 (3.0) | 0 (0.0) | 0 (0.0) | 0 (0.0) | 1050 (34.9) | 0 (0.0) | 1352 (44.9) |
|  |  |  | II | 4052 (4.0) | 3 (0.1) | 3 (0.1) | 0 (0.0) | 3410 (84.2) | 0 (0.0) | 1212 (29.9) |
|  |  |  | III | 1012 (1.0) | 13 (1.3) | 7 (0.7) | 6 (0.6) | 935 (92.4) | 0 (0.0) | 742 (73.3) |
|  |  |  |  |  |  |  |  |  |  |  |
| ER- | HER2+ | < 50 | I | 345 (0.3) | 253 (73.3) | 41 (11.9) | 212 (61.4) | 16 (4.6) | 244 (70.7) | 155 (44.9) |
|  |  |  | II | 516 (0.5) | 498 (96.5) | 60 (11.6) | 438 (84.9) | 33 (6.4) | 468 (90.7) | 265 (51.4) |
|  |  |  | III | 291 (0.3) | 286 (98.3) | 33 (11.3) | 253 (86.9) | 18 (6.2) | 262 (90.0) | 262 (90.0) |
|  |  | 50-75 | I | 1182 (1.2) | 583 (49.3) | 101 (8.5) | 482 (40.8) | 29 (2.5) | 541 (45.8) | 679 (57.4) |
|  |  |  | II | 1193 (1.2) | 984 (82.5) | 172 (14.4) | 812 (68.1) | 50 (4.2) | 926 (77.6) | 605 (50.7) |
|  |  |  | III | 492 (0.5) | 444 (90.2) | 70 (14.2) | 374 (76) | 12 (2.4) | 416 (84.6) | 441 (89.6) |
|  |  | >75 | I | 81 (0.1) | 0 (0) | 0 (0.0) | 0 (0.0) | 2 (2.5) | 0 (0.0) | 23 (28.4) |
|  |  |  | II | 202 (0.2) | 8 (4) | 2 (1.0) | 6 (3.0) | 8 (4.0) | 8 (4.0) | 54 (26.7) |
|  |  |  | III | 110 (0.1) | 9 (8.2) | 5 (4.5) | 4 (3.6) | 7 (6.4) | 8 (7.3) | 75 (68.2) |
|  |  |  |  |  |  |  |  |  |  |  |
| ER- | HER2- | < 50 | I | 1363 (1.3) | 1055 (77.4) | 508 (37.3) | 546 (40.1) | 40 (2.9) | 0 (0.0) | 903 (66.3) |
|  |  |  | II | 1830 (1.8) | 1747 (95.5) | 777 (42.5) | 970 (53) | 120 (6.6) | 12 (0.7) | 1155 (63.1) |
|  |  |  | III | 375 (0.4) | 362 (96.5) | 114 (30.4) | 248 (66.1) | 37 (9.9) | 7 (1.9) | 356 (94.9) |
|  |  | 50-75 | I | 3286 (3.2) | 1327 (40.4) | 667 (20.3) | 660 (20.1) | 59 (1.8) | 9 (0.3) | 2373 (72.2) |
|  |  |  | II | 2790 (2.8) | 2053 (73.6) | 1071 (38.4) | 982 (35.2) | 163 (5.8) | 13 (0.5) | 1657 (59.4) |
|  |  |  | III | 709 (0.7) | 589 (83.1) | 234 (33) | 355 (50.1) | 41 (5.8) | 6 (0.8) | 619 (87.3) |
|  |  | >75 | I | 303 (0.3) | 3 (1.0) | 2 (0.7) | 1 (0.3) | 7 (2.3) | 0 (0.0) | 122 (40.3) |
|  |  |  | II | 719 (0.7) | 11 (1.5) | 6 (0.8) | 5 (0.7) | 36 (5.0) | 0 (0.0) | 201 (28.0) |
|  |  |  | III | 267 (0.3) | 9 (3.4) | 5 (1.9) | 4 (1.5) | 18 (6.7) | 0 (0.0) | 187 (70.0) |
| Missing |  |  |  | 1433 (1.4) | 244 (17) | 99 (6.9) | 145 (10.1) | 272 (19) | 62 (4.3) | 799 (55.8) |

Abbreviations: ER = oestrogen receptor, HER2 = human epidermal growth factor receptor 2

*V3.1: standard-dose anthracycline = generation 2; high-dose anthracycline/taxane-based = generation 3.

**Supplementary Table 3**. Baseline characteristics and 10-year survival in the not adjuvant treated patients of the Dutch validation cohort.

| **Characteristic** | **n = 12,019** |
| --- | --- |
| Year of diagnosis | 2005-2013 |
| Age, median (IQR) | 66.0 (55.0-74.0) |
| Menopausal status, n (%)*  Pre-menopausal  Post-menopausal  Missing | 453 (14.9)  2,594 (851)  8,972 |
| Tumour size (mm), median (IQR)  Missing | 12.0 (8.0 – 11.0)  468 |
| Lymph nodes, n (%)  ≥ 1  0  Missing | 1,302 (11.2%)  10,288 (88.8%)  429 |
| Tumour stage, n (%)  Stage I  Stage II  Stage III | 9241 (76.9)  2,495 (20.8)  283 (2.4) |
| Differentiation, n (%)  Grade 1  Grade 2  Grade 3  Missing | 4,282 (38.6)  4,543 (40.9)  2,276 (20.5)  918 |
| Oestrogen receptor status, n (%)  Positive  Negative  Missing | 9,012 (78.2)  2,516 (21.8)  491 |
| HER2 status, n (%)  Positive  Negative  Unknown  Missing | 1,249 (10.4)  9,384 (78.4)  1,342 (11.2)  44 |
| Progesterone status, n (%)  Positive  Negative  Unknown  Missing | 7,022 (58.6)  4,204 (35.1)  747 (6.2)  46 |
| Mode of detection, n (%)  Clinically detected  Screening detected  Missing | 1,383 (52.4)  1,258 (47,6)  9,378 |
| Vital status at 10-year, n (%)  Alive  Death | 7,984 (66.4)  4,035 (33.6) |
|  |  |

Abbreviations: IQR = interquartile range, HER2 = human epidermal growth factor receptor 2

*Menopausal status was used to determine eligibility for bisphosphonate therapy and was not included as predictor in the model

**Supplementary Table 4**. Observed and predicted 10-year overall survival for patients who did not receive adjuvant therapy by subgroups based on ER status, HER2 status, age, and tumour stage for PREDICT v2.2 and v3.1.

|  | | | |  |  | **PREDICT v2.2** | | **PREDICT v3.1** | |
| --- | --- | --- | --- | --- | --- | --- | --- | --- | --- |
|  |  |  |  | N (%) | Observed, % (95% CI) | Predicted, % | Difference, % | Predicted, % | Difference, % |
| **Total cohort** | | | | 12019 (100.0) | 66.4 (65.6-67.3) | 70.5 | 4.1 | 73.1 | 6.7 |
|  | | | | | | | | | |
| **ER-status** | **HER2-status** | **Age** | **Stage** |  |  |  |  |  |  |
| ER+ | HER2+ | < 50 | I | 89 (0.7) | 93.3 (88.2-98.6) | 89.6 | -3.6 | 90.6 | -2.7 |
|  |  |  | II | 15 (0.1) | 73.3 (54.0-99.5) | 77.7 | 4.4 | 77.4 | 4.1 |
|  |  |  | III | - | - | - | - | - | - |
|  |  | 50-75 | I | 341 (2.8) | 77.1 (72.8-81.7) | 80.8 | 3.6 | 82.6 | 5.5 |
|  |  |  | II | 42 (0.3) | 47.6 (34.7-65.4) | 64.1 | 16.5 | 65.2 | 17.6 |
|  |  |  | III | 7 (0.1) | 14.3 (2.3-87.7) | 28.0 | 13.7 | 30.7 | 16.5 |
|  |  | >75 | I | 42 (0.3) | 40.5 (28.0-58.4) | 45.8 | 5.3 | 49.9 | 9.4 |
|  |  |  | II | 25 (0.2) | 32.0 (18.1-56.7) | 32.9 | 0.9 | 31.8 | -0.2 |
|  |  |  | III | 4 (0.0) | 25.0 (4.6-100.0) | 18.6 | -6.4 | 18.7 | -6.3 |
|  |  |  |  |  |  |  |  |  |  |
| ER+ | HER2- | < 50 | I | 1122 (9.3) | 92.2 (90.6-93.7) | 92.3 | 0.2 | 93.4 | 1.2 |
|  |  |  | II | 143 (1.2) | 84.6 (78.9-90.7) | 86.3 | 1.7 | 87.3 | 2.7 |
|  |  |  | III | 9 (0.1) | 22.2 (6.5-75.4) | 44.2 | 22.0 | 45.5 | 23.3 |
|  |  | 50-75 | I | 5331 (44.4) | 79.9 (78.8-81.0) | 82.2 | 2.3 | 85.1 | 5.2 |
|  |  |  | II | 641 (5.3) | 63.7 (60.0-67.5) | 73.9 | 10.3 | 75.3 | 11.6 |
|  |  |  | III | 43 (0.4) | 25.6 (15.4-42.6) | 40.4 | 14.8 | 42.6 | 17.0 |
|  |  | >75 | I | 1076 (8.9) | 38.6 (35.8-41.6) | 47.2 | 8.7 | 52.0 | 13.4 |
|  |  |  | II | 493 (4.1) | 21.7 (18.4-25.7) | 39.9 | 18.2 | 42.3 | 20.6 |
|  |  |  | III | 49 (0.4) | 4.1 (1.1-15.9) | 23.2 | 19.1 | 21.9 | 17.8 |
|  |  |  |  |  |  |  |  |  |  |
| ER- | HER2+ | < 50 | I | 57 (0.5) | 77.2 (67.0-88.9) | 77.9 | 0.7 | 84.3 | 7.2 |
|  |  |  | II | 11 (0.1) | 54.5 (31.8-93.6) | 57.4 | 2.9 | 59.7 | 5.1 |
|  |  |  | III | 4 (0.0) | 50.0 (18.8-100.0) | 19.5 | -30.5 | 13.9 |  |
|  |  | 50-75 | I | 279 (2.3) | 76.3 (71.5-81.5) | 67.3 | -9.1 | 76.4 | 0.1 |
|  |  |  | II | 118 (1.0) | 45.8 (37.6-55.7) | 45.3 | -0.5 | 45.7 | -0.1 |
|  |  |  | III | 20 (0.2) | 15.0 (5.3-42.6) | 15.0 | 0.0 | 12.8 | -2.2 |
|  |  | >75 | I | 60 (0.5) | 30.0 (20.4-44.2) | 35.2 | 5.2 | 37.9 | 7.9 |
|  |  |  | II | 144 (1.2) | 25.7 (19.5-33.9) | 25.5 | -0.2 | 21.5 | -4.2 |
|  |  |  | III | 34 (0.3) | 5.9 (1.5-22.6) | 8.2 | 2.3 | 6.3 | 0.4 |
|  |  |  |  |  |  |  |  |  |  |
| ER- | HER2- | < 50 | I | 110 (0.9) | 88.2 (82.4-94.4) | 83.2 | -5.0 | 87.4 | -0.8 |
|  |  |  | II | 38 (0.3) | 57.9 (44.1-75.9) | 70.0 | 12.1 | 65.4 | 7.5 |
|  |  |  | III | 6 (0.0) | 33.3 (10.8-100.0) | 37.4 | 4.1 | 30.4 | -2.9 |
|  |  | 50-75 | I | 560 (4.7) | 69.1 (65.4-73.0) | 70.2 | 1.1 | 77.4 | 8.3 |
|  |  |  | II | 352 (2.9) | 46.3 (41.4-51.8) | 54.1 | 7.8 | 55.2 | 8.9 |
|  |  |  | III | 36 (0.3) | 16.7 (8.0-34.6) | 33.4 | 16.7 | 32.7 | 16.0 |
|  |  | >75 | I | 174 (1.4) | 27.0 (21.2-34.5) | 39.7 | 12.7 | 43.3 | 16.3 |
|  |  |  | II | 473 (3.9) | 23.5 (19.9-27.6) | 30.9 | 7.5 | 28.9 | 5.4 |
|  |  |  | III | 71 (0.6) | 9.9 (4.9-19.9) | 13.6 | 3.8 | 10.1 | 0.2 |

Abbreviations: N = total number, CI = confidence interval, ER = oestrogen receptor, HER2 = human epidermal growth factor receptor 2

**Supplementary Figure 1**. Observed and predicted 10-year overall survival for patients that did not receive adjuvant therapy of PREDICT v2.2 and v3.1 with the 95% confidence interval.


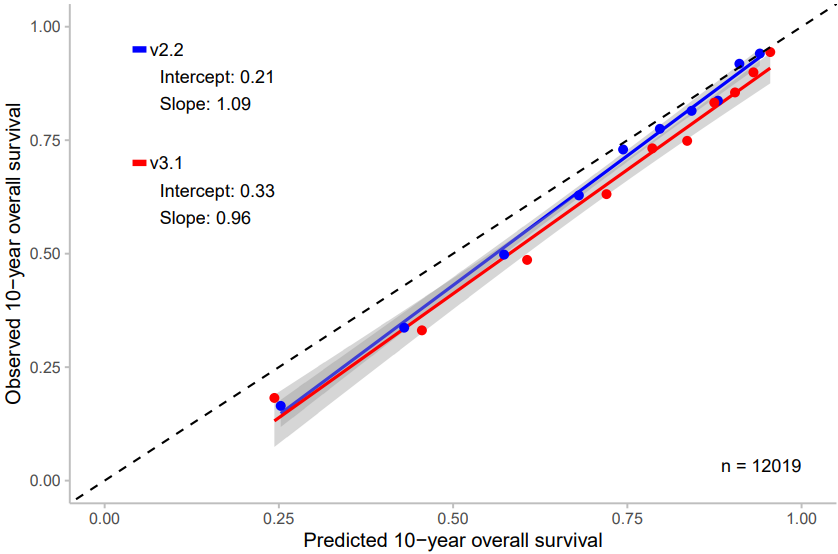


*n = number of patients included in the external validation set. Calibration is assessed using the intercept (ideally 0) and slope (ideally 1). The Y-axis represents the observed 10-year overall survival, while the X-axis represents the predicted 10-year overall survival. Each dot indicate the average for a decile of predicted probabilities. In grey the 95% confidence intervals are shown.*
